# Rapid changes in neuroendocrine regulation may contribute to reversal of type 2 diabetes after gastric bypass surgery

**DOI:** 10.1007/s12020-020-02203-w

**Published:** 2020-01-26

**Authors:** Petros Katsogiannos, Prasad G. Kamble, Urban Wiklund, Magnus Sundbom, Daniel Espes, Ulf Hammar, F. Anders Karlsson, Maria J. Pereira, Jan W. Eriksson

**Affiliations:** 1 Department of Medical Science, Clinical Diabetes and Metabolism, Uppsala University, Uppsala, Sweden; 2 Radiation Sciences, Biomedical Engineering & Informatics, Umeå University, Umeå, Sweden; 3 Surgical Sciences, Uppsala University, Uppsala, Sweden; 4 Department of Medical Cell Biology, Uppsala University, Uppsala, Sweden

**Keywords:** Roux-en-Y gastric bypass, Type 2 diabetes, Incretins, Adipokines, Heart rate variability

## Abstract

**Objective:**

To explore the role of hormones and the autonomic nervous system in the rapid remission of diabetes after Roux-en-Y Gastric Bypass (RYGB).

**Research design and methods:**

Nineteen obese patients with type 2 diabetes, 7 M/12 F, were randomized (2:1) to RYGB or standard-of-care medical treatment (control). At baseline and 4 and 24 weeks post surgery, fasting blood sampling, OGTT, intravenous arginine challenge, and heart-rate variability (HRV) assessments were performed.

**Results:**

At both 4 and 24 weeks post-RYGB the following effects were found: arginine-stimulated insulin secretion was reduced. GLP-1, GIP, and glucagon rise during OGTT was enhanced. IGF-1 and GH levels increased. In addition, total HRV and spectral components *P*_LF_ (power of low frequency) and *P*_HF_ (power of high frequency) increased. At 4 weeks, morning cortisol was lower than baseline and 24 weeks. At 24 weeks, NEFA levels during OGTT, and the *P*_LF_/*P*_HF_ ratio decreased. None of these changes were seen in the control group.

**Conclusions:**

There were rapid changes within 4 weeks after RYGB: signs of enhanced parasympathetic nerve activity, reduced morning cortisol, and enhanced incretin and glucagon responses to glucose. The findings suggest that neurohormonal mechanisms can contribute to the rapid improvement of insulin resistance and glycemia following RYGB in type 2 diabetes.

## Introduction

Bariatric surgery, in particular, Roux-en-Y Gastric Bypass (RYGB), markedly improves glycemic control and can prevent or reverse type 2 diabetes in obese individuals [1]. The metabolic effects appear to be partly independent of weight loss, occurring earlier than weight loss, and with no apparent correlation to its magnitude. The underlying mechanisms remain unclarified [2]. Multiple pathways are proposed to be involved in these effects of RYGB, including altered route and timing of food delivery to the small intestine, adipose morphology and function, hepatic glucose turnover, CNS control of nutrient intake and metabolism, as well as various hormones [3].

Following RYGB, there are substantial changes in gut hormones including elevated or faster responses of glucagon-like peptide-1 (GLP-1), glucose-dependent insulinotropic peptide (GIP), and peptide YY (PYY) in the postprandial state. This is also seen for glucagon [4, 5].

Rapid delivery of ingested glucose into the small intestine’s distal part contributes to the increased GLP-1 response promoting insulin secretion [6]. This, together with GLP-1 effects in the CNS leading to reduced food intake [7], may partly explain improved glucose tolerance after RYGB.

The autonomic nervous system (ANS) may be of importance for the metabolic effects of RYGB. There may be a transient activation of the sympathetic nervous system and subsequently a relative increase in parasympathetic over sympathetic activity [8]. Importantly, with RYGB the stomach and its vagus branches are cut transversely, and thus afferent vagal pathways to the CNS are affected [9].

Our cohort consists of obese patients with type 2 diabetes, randomized 2:1 to RYGB or conventional diabetes treatment. In a first report, we showed that insulin action in adipocytes was not improved after 4 weeks, despite markedly reduced cell size, and could not explain the short-term improvement of whole-body insulin sensitivity [10]. Here we report rapid changes in hormonal and autonomic nerve regulation which can contribute to the antidiabetic effects of RYGB.

## Research design and methods

### Patient selection and study design

We included patients with type 2 diabetes aged 18–60 with BMI between 30 and 45 kg/m^2^. The detailed eligibility criteria, anthropometric, and clinical characteristics of participants were recently reported [10]. Patients were randomly assigned 2:1 to RYGB or standard-of-care medical treatment. The surgery group had 13 patients (10 F) whereas 6 patients (2 F) were allocated to the control group. Due to the nature of the intervention, blinding was not feasible for patients and staff. The Regional Ethics Review Board in Uppsala approved the study, and all participants gave their written informed consent before enrolment (ClinicalTrials.gov Identifier: NCT02729246).

### Study procedures

Patients randomized to surgical intervention (RYGB group) were assessed on five occasions; at baseline, following a 4-week low-calorie diet (LCD; 800–1100 kcal/day) in the morning just before surgery, at 4 and 24 weeks and (not reported here) 2 years after laparoscopic RYGB. The control group received routine lifestyle advice and continued or adjusted medications according to clinical guidelines. Investigations were carried out at baseline and after 24 weeks. The following investigations were performed during 1-day visits in both groups: anthropometric measurements (weight, waist/hip circumference, and body fat% using bioimpedance), 5-minute heart-rate variability (HRV) registration, fasting blood tests, and a needle biopsy of abdominal subcutaneous adipose tissue. Insulin secretion was assessed after administrating a bolus dose of 5 g arginine iv, followed by measuring S-insulin at 0, 2, 3, 4, 5, 7, 10, 25, and 30 minutes [11]. Lastly, a 3 h oral glucose tolerance test (OGTT, 75 g glucose) during which hormones were measured and the Matsuda and insulinogenic indices were calculated as a measure of insulin sensitivity and secretion, respectively [12]. These procedures were not performed at the visit after the LCD diet.

After RYGB, antidiabetic medications were reduced or withdrawn as judged clinically appropriate and other medication changes were made when medically mandated.

### Biochemical measurements and statistical analyses

These methods are described in Supplementary material.

## Results

Beneficial metabolic effects in RYGB patients, including weight loss and improved glycemic control and insulin sensitivity, were recently published [10]. In summary, the patients undergoing surgery showed significant reductions after 4 and 24 weeks in BMI, fasting insulin levels, and HbA1c with the reductions being more pronounced after 24 weeks. All but one of the patients discontinued their diabetes medication (Table 1).

**Table 1:** List of medications in the surgical group

	Baseline	Post-LCD	4 weeks post surgery	24 weeks post surgery
Metformin (average dose 1700 ± 888 mg)	11	11	1^a^	1^a^
Other OAD (DPP-4 inhibitor *n* = 2) (GLP-1 analog *n* = 1)	3	3	0	0
Lipid-lowering drug (simvastatin *N* = 6, avg. dose 20 ± 11 mg; atorvastatin *N* = 4 avg. dose 17.5 ± 5 mg)	10	10	0	0
Antihypertensive drug ACE inhibitor/ARB *n* = 7 Betablocker *n* = 3 Calcium antagonist *n* = 4 Diuretic thiazide *n* = 1	8	8	1	0

One patient was on diet therapy only

^a^The patient that continued treatment with metformin after RYGB received a lower dose

### Incretin hormones

**Fig. 1:**
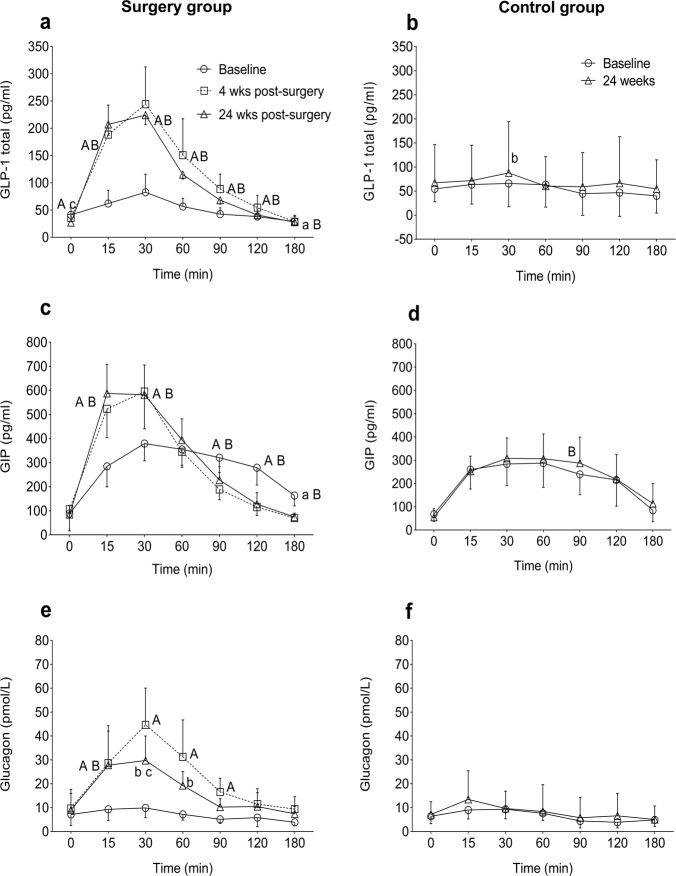
Plasma incretins and glucagon in response to OGTT in surgery and control groups. **a** GLP-1 in surgery group; **b** GLP-1 in control group; **c** GIP in surgery group; **d** GIP in control group; **e** glucagon in surgery group; **f** glucagon in control group. Data are shown as mean ± 95% CI. ^a,A^Baseline vs post surgery at 4 weeks. ^b,B^Baseline vs post surgery at 24 weeks. ^c,C^4 weeks vs post surgery at 24 weeks. Lower case letters; *p* < 0.05, upper case letters; *p* < 0.01

Total GLP-1 and GIP levels were measured in plasma during fasting and OGTT at different visits (Table 2, Fig. 1). In the surgery group, fasting GLP-1 levels decreased at 4 weeks (*p* = NS) and 24 weeks (*p* < 0.01) compared with baseline (Table 2). GLP-1 response during OGTT was higher at both 4 and 24 weeks post surgery compared with baseline (Fig. 1a). The AUC for GLP-1 was higher at 4 and 24 weeks (Table 3, *p* < 0.05 for both visits). In the control group, GLP-1 levels at fasting and during OGTT did not change between visits (Fig. 1b).

**Table 2:** Plasma fasting concentration of hormones, metabolic substrates, and adipokines in the surgery and control group

Variable	Surgery group (*n* = 13)			Control group (*n* = 6)	
	Baseline	4 weeks post surgery	24 weeks post surgery	Baseline	24 weeks
Body weight (kg)	100 (14)	89 (11)^A^	77 (10)^B,C^	116 (8)	115 (9)
BMI (kg/m^2^)	36.8 (4.0)	32.7 (3.2)^A^	28.5 (3.2)^B,C^	36.2 (4)	35.7 (4.3)
Fasting insulin (mU/L)	23.5 (10.5)	10.2 (5.1)^A^	5.8 (1.6)^B,C^	23.2 (16.4)	26.7 (26.4)
AIR_arg_ (mU/ml)	66 (32)	37 (19)^A^	34 (18)^B^	75 (48)	102 (99)
Matsuda index	1.4 (0.5)	2.9 (1.2)^A^	4.4 (1.9)^B,c^	2.7 (2.5)	2.9 (3.1)
GLP-1 (total) (pg/ml)	43 (23)	36 (18)	27 (17)^B,c^	55 (22)	67 (64)
GIP (total) (pg/ml)	89 (32)	106 (134)	85 (49)	68 (29)	55 (34)
Glucagon (pmol/L)	8 (8)	10 (10)	10 (13)	6 (3)	7 (5)
Cortisol (nmol/L)	265 (81)	217 (59)^a^	282 (71)^c^	225 (44)	206 (32)
IGF1 (µg/L)	129 (65)	137 (65)	154 (73)^B,c^	144 (64)	172 (76)
Growth hormone (µg/L)	0.37 (0.39)	0.80 (0.76)	2.29 (1.86)^B,C^	NA	NA
NEFA (µmol/L)	243 (73)	240 (71)	198 (68)	206 (99)	225 (112)
Glycerol (µmol/L)	116 (79)	87 (60)^a^	103 (93)	143 (51)	209 (120)
Adiponectin (ng/ml)	4924 (2474)	5059 (2740)	7262 (3329)^B,c^	NA	NA
Leptin (pg/ml)	78684 (967378)	24660 (24531)^a^	15119 (11259)^b^	NA	NA
Visfatin (pg/ml)	1879 (635)	1632 (680)	2646 (1208)^b,C^	NA	NA
Resistin (pg/ml)	6133 (1779)	6577 (1136)	7066 (1961)^b^	NA	NA
BMP4 (pg/ml)	19 (3)	19 (3)	21 (3)^b,C^	NA	NA

Data are shown as mean (SD). Lower case letters; *p* < 0.05, upper case letters; *p* < 0.01. ^a,A^Baseline vs treatment at 4 weeks. ^b,B^Baseline vs treatment at 24 weeks. ^c,C^4 weeks vs treatment at 24 weeks

*NA* no analysis was done, *AIR*_*arg*_ acute insulin response after i.v. arginine, *BMI* body mass index, *AIRarg* acute insulin response during arginine test, *GLP-1* glucagon-like peptide-1, *GIP* glucose-dependent insulinotropic peptide, *IGF1* insulin-like growth factor 1, *NEFA* non-esterified fatty acids, *BMP4* Bone morphogenic protein-4

**Table 3:** Area under the curve for hormones and metabolic substrates measured during OGTT between surgery and control group

Variable	Surgery group (*n* = 13)			Control group (*n* = 6)	
	Baseline	4 weeks post surgery	24 weeks post surgery	Baseline	24 weeks
GLP-1 AUC 180 min (min × pmol/L)	8310 (5770–10930)	16540 (14664–24603)^a^	15936 (14756–18827)^b^	6193 (3879–10468)	6119 (3619–11498)
GIP AUC 180 min (min × pg/ml)	50524 (38646–58457)	42381 (40111–51209)	51220 (37331–59300)	38446 (28960–48365)	43743 (36483–48192)
Glucagon AUC 180 min (min × pmol/L)	1127 (761–1567)	4104 (2439–5150)^A^	2758 (2441–3247)^b^	1025 (894–1346)	800 (519–1231)
NEFA AUC 180 min (min × μmol/L)	24185 (22004–30204)	24012 (21549–27315)	19367 (16869–23511)	20814 (16624–26739)	19748 (12108–30595)
Glycerol AUC 180 min (min × μmol/L)	17949 (13659–26723)	13637 (8380–28147)	10410 (8684–19164)	18536 (13966–24124)	23018 (18189–32369)
Insulin_Arg_ AUC 30 min (min × mU/L)	1215 (974–1596)	665 (578–822)	583 (374–646)	1376 (419–1757)	1189 (449–2353)

Data are shown as median (25th–75th percentile). Lower case letters; *p* < 0.05, upper case letters; *p* < 0.01. ^a,A^Baseline vs treatment at 4 weeks. ^b,B^Baseline vs treatment at 24 weeks

*GLP-1* glucagon-like peptide-1, *GIP* glucose-dependent insulinotropic peptide, *NEFA* nonesterified fatty acids, insulin measured during iv arginine challenge

In the surgery group, fasting GIP levels did not differ between visits (Table 2). The peak GIP levels during OGTT (30 min) were higher at 4 and 24 weeks. (Fig. 1c, *p* < 0.01 for both visits), and instead, a steep decline was observed after 30 min. The total AUC for GIP during OGTT were similar between visits (Table 3). In the control group, neither fasting nor glucose-induced GIP secretion differed between visits (Fig. 1d).

### Glucagon

In the surgery group, fasting glucagon levels did not differ between visits (Table 2, Fig. 1e). At the baseline visit, no change in glucagon levels was observed during OGTT (Fig. 1e). However, at 4 and 24 weeks, glucagon levels were significantly higher during OGTT than baseline, which then dropped to their starting levels at 180 min, but remained numerically higher than the baseline visit. Peak glucagon levels at 4 weeks during OGTT were higher than at 24 weeks (*p* < 0.05). The total AUC for glucagon during OGTT was higher at 4 and 24 weeks compared with baseline (Table 3, *p* < 0.01 and *p* < 0.05 for 4 and 24 weeks, respectively). In the control group, glucagon levels both fasting and during OGTT did not change between visits (Fig. 1f).

### Cortisol

In the surgery group, morning cortisol levels were lower at 4 weeks compared with baseline (*p* < 0.05). At 24 weeks, cortisol had returned to baseline levels. In the control group, no change in the fasting cortisol levels was observed (Table 2).

### IGF1 and growth hormone

As shown in Table 2, fasting IGF1 levels at 24 weeks were higher than baseline (*p* < 0.01) and 4 weeks after surgery (*p* < 0.05). In the surgery group, growth hormone levels increased numerically at 4 (*p* = 0.065) and significantly at 24 weeks (*p* < 0.01) compared with baseline.

### Insulin secretion during arginine challenge

**Fig. 2:**
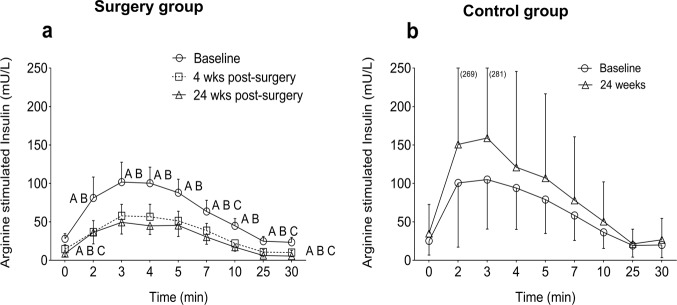
Arginine-stimulated insulin secretion in surgery and control groups. **a** Insulin levels during arginine challenge in surgery group, **b** insulin levels during arginine challenge in control group. Data are shown as mean ± 95% CI. ^A^Baseline vs post surgery at 4 weeks. ^B^baseline vs post surgery at 24 weeks. ^C^4 weeks vs post surgery at 24 weeks. Lower case letters; *p* < 0.05, upper case letters; *p* < 0.01

Insulin secretion during arginine challenge decreased at 4 and 24 weeks compared with baseline (Fig. 2a). The total AUC for insulin during the arginine challenge was lower post surgery compared with baseline (by about 50%, *p* < 0.01) (Table 3). The acute insulin response to arginine at the three peak time points (2, 3, and 4 min) was lower by 45% (*p* < 0.01) and 48% (*p* < 0.01) at 4 and 24 weeks, respectively, compared with baseline (Table 2). However, the relative increase in peak insulin levels after arginine did not change at 4 weeks but was higher by 85% (*p* < 0.05) at 24 weeks compared with baseline. In the control group, there were no significant changes over time (Fig. 2b).

### Circulating levels of adipokines

Data are shown in Table 2. At 24 weeks, adiponectin levels were higher than baseline (*p* < 0.01) as well as 4 weeks (*p* < 0.05). Leptin levels reduced at 4 and 24 weeks after surgery (*p* < 0.05 for both visits). An additional reduction in leptin levels was observed at 24 weeks (*p* = 0.053). At 24 weeks, visfatin levels were higher than baseline (*p* < 0.05) and 4 weeks (*p* < 0.01). Likewise, plasma resistin was higher at 24 weeks than baseline (*p* < 0.05). At 24 weeks, BMP4 levels were higher than baseline (*p* < 0.05) and 4 weeks (*p* < 0.01).

### NEFA and glycerol

**Fig. 3:**
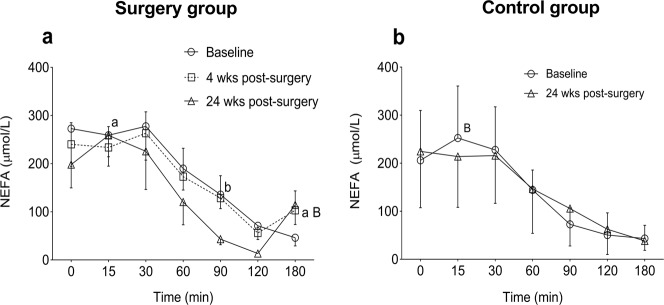
NEFA and glycerol levels during OGTT in surgery and control group. **a** NEFA in surgery group; **b** NEFA in control group. Data are shown as mean ± 95% CI. ^a,A^Baseline vs post surgery at 4 weeks. ^b,B^Baseline vs post surgery at 24 weeks. Lower case letters; *p* < 0.05, upper case letters; *p* < 0.01

In the surgery group, NEFA levels during OGTT did not differ between baseline and 4 weeks but remained numerically lower at 24 weeks than both other visits (Fig. 3a). There was a rise in NEFA between 120 and 180 min. At 180 min, the NEFA levels at 4 (*p* < 0.05) and 24 weeks (*p* < 0.01) were higher than baseline. The total AUC for NEFA during OGTT did not significantly change between visits (Table 3). Fasting glycerol levels were lower at 4 weeks compared with baseline (*p* < 0.05, Table 2). However, during OGTT, no change in the glycerol levels was observed between visits (data not shown). In the control group, both NEFA and glycerol levels at fasting and during OGTT did not change between visits (Table 3, Fig. 3b and data not shown).

### Heart-rate variability

Table 4 summarizes HRV data. When comparing baseline, HRV at 4 and 24 weeks post surgery were significantly increased. The RR interval was longer, corresponding to decreased heart rate at 4 and 24 weeks post surgery, compared with baseline. There were significant rises in *P*_tot_ (total variability measurement) and spectral components such as *P*_VLF_ (represent sympatho-vagal balance), *P*_LF_, and *P*_HF_ at 4 and 24 weeks post surgery compared with baseline. *P*_LF_/*P*_HF_ ratio, a measure of the balance between sympathetic and parasympathetic activity, did not change 4 weeks post surgery compared with baseline but was significantly decreased at 24 weeks post surgery. The change in the Matsuda index from baseline to 4 weeks after surgery was positively associated with the change in *P*_tot_ (rho = 0.762, *p* < 0.05) and *P*_LF_ (rho = 0.893, *p* < 0.01) at 4 weeks after surgery. There were no associations between change in the Matsuda index at 24 weeks and a change in HRV measures at 4 or 24 weeks.

**Table 4:** Heart-rate variability

	Surgery group			Control group	
		Change from baseline			Change from baseline
Variable	Baseline (*n* = 10)	4 weeks post surgery (*n* = 8)	24 weeks post surgery (*n* = 10)	Baseline (*n* = 6)	24 weeks (*n* = 6)
RR (s)	0.81 (0.13)	0.16 (0.09)^A^	0.18 (0.06)^B^	0.86 (0.13)	−0.04 (0.05)
*P*_tot_ (ms^2^, log)	2.93 (0.46)	0.22 (0.21)^a^	0.36 (0.35)^B^	2.98 (0.51)	0.02 (0.18)
*P*_VLF_ (ms^2^, log)	2.59 (0.50)	0.18 (0.30)	0.28 (0.38)^b^	2.69 (0.66)	0.08 (0.36)
*P*_LF_ (ms^2^, log)	2.43 (0.42)	0.21 (0.24)^a^	0.30 (0.40)^b^	2.46 (0.48)	0.01 (0.19)
*P*_HF_ (ms^2^, log)	2.04 (0.67)	0.29 (0.26)^a^	0.59 (0.46)^B^	1.98 (0.42)	−0.16 (0.21)
*P*_LF_/*P*_HF(log)_	0.39 (0.32)	−0.09 (0.29)	−0.28 (0.30)^b^	0.48 (0.28)	0.17 (0.17)

Data are means (SD) of baseline values and of changes between follow-ups and baseline. Spectral Indices are log-transformed. RR = mean RR interval, *P*_tot_ = total power, *P*_VLF_ = power of very low-frequency component, *P*_LF_ = power of low-frequency component, *P*_HF_ = power of high-frequency component. Lower case letters; *p* < 0.05, upper case letters; *p* < 0.01 (paired *t-*tests). ^a,A^Change at 4 weeks from baseline. ^b,B^Change at 24 weeks from baseline

In the control group, no significant changes were observed in any of the above measures.

## Discussion

We present a comprehensive and integrated assessment of changes in hormonal, metabolic, and ANS regulation at 4 and 24 weeks post-RYGB. Novel findings include the following: HRV, in particular, the high-frequency (HF) component, was rapidly increased, within 4 weeks after RYGB suggesting an enhanced parasympathetic outflow. Later on, there were signs of attenuation of sympathetic outflow (reduced LF/HF ratio). Morning cortisol was decreased at 4 weeks. Other post-RYGB changes were seen later on, at 24 weeks, when plasma levels of adiponectin, visfatin, BMP4, and resistin were increased. At this time leptin was reduced and so were NEFA levels during OGTT.

As we reported previously [10] glucose and insulin levels reached a peak earlier during OGTT both at 4 and 24 weeks after surgery. However, glucose and insulin at 120 min of OGTT were lower. The AUC during OGTT for glucose and insulin was lower at 4 and 24 weeks and this indicates an increased insulin sensitivity. The insulin response to iv arginine challenge was markedly lower post-RYGB. Therefore, the increased early response during OGTT suggest important effects of incretins and other insulinotropic gut factors, that contribute to the insulin rapid response during oral (but not IV) nutrient exposure.

Markedly elevated responses of incretin hormones and glucagon to oral glucose ingestion were seen 4 weeks after surgery, and whole-body insulin sensitivity rapidly improved post surgery, within 4 weeks.

There is rapid improvement, within a few days, of insulin resistance and glycemic control in obese patients with type 2 diabetes or prediabetes following RYGB [13]. Since this occurs before any significant body weight loss, reduction or remodeling of adipose tissue is probably not critical for the early glycemic effects [10]. Instead, changes in hormone secretion, nutrient fluxes, or ANS activity might contribute to improved insulin sensitivity after RYGB.

### Incretins

We demonstrate that plasma GLP-1 and GIP levels rose markedly more during OGTT at 4 and 24 weeks after RYGB compared with baseline. The time for peak GLP-1 levels was 30 min after glucose ingestion, similar to normal individuals [14]. Our results are in agreement with previous studies in obese, as well as lean, type 2 diabetes patients that reported increased GLP-1 levels during OGTT about 1 month after RYGB [15, 16]. Moreover, a study in obese type 2 diabetes patients showed elevated levels of GLP-1 after repeated meal tests at 7, 30, and 90 days after RYGB [17]. In contrast to GLP-1, the findings related to GIP are less consistent. The studies have shown either increased, decreased, or unchanged in GIP levels after RYGB [15]. We found a more rapid and enhanced GIP response to OGTT which is in accordance with a previous study [18]. The different results for GIP levels could be explained by different patient phenotypes and analytical methods between studies.

The mechanisms of the rise in incretin levels after bariatric surgery are not entirely understood. However, the surgical procedure of RYGB which bypasses the upper gut, making the lower gut exposed to nutrients soon after ingestion may potentially change the timing of the release of incretin hormones [19]. Although cells producing GLP-1 and GIP are present in different location in gastrointestinal tract, the peak levels for both these incretin hormones were achieved by 30 min after ingestion of oral glucose, which may suggest that the rapid food entry into the intestine is not critical.

### Glucagon

Type 2 diabetes patients generally have hyperglucagonemia, in particular, postprandially, and this is considered to contribute to hyperglycemia after meals. We observed a marked increase in the glucagon response during OGTT after surgery, and yet postprandial glucose levels were reduced. The glucagonostatic properties of GLP-1 and its exaggerated response after RYGB would predict a reduced release of glucagon as observed after a meal [20]. Nonetheless, our results agree with previous reports showing a rise in glucagon during OGTT, in obese women with type 2 diabetes 1 month after RYGB, despite an increase in GLP-1 levels [21]. Eliminating the effect of endogenous GLP-1 with Exendin-9 further increased glucagon secretion, implying that meal-induced glucagon release continues to be under the inhibitory control of GLP-1 [22]. It could be speculated that L-cells could at least transiently secrete some glucagon due to altered cleavage of proglucagon, yielding not only GLP-1 and 2 and glicentin but also glucagon. In fact, extrapancreatic glucagon release has been demonstrated previously, and glucagon production in the small intestine was recently reported [23].

This could be due to L-cell stress due to overwhelming nutrient stimuli during meals and might be analogous to beta-cell stress in type 2 diabetes, leading to an increased plasma proinsulin/insulin ratio due to insufficient proconvertase activity [24]. Nonetheless, the mechanisms underlying the increased meal-induced glucagon response remain to be elucidated.

### Adipokines

Leptin is an adipose-derived hormone involved in long-term energy balance, mainly by limiting food intake via effects in the hypothalamus. Circulating leptin levels correlate with body fat mass [25]. We found significantly reduced fasting leptin levels 4 weeks post surgery with an additional reduction at 24 weeks. The reduction in leptin levels probably reflects the negative balance of lipid, i.e. energy, stores of adipose tissue as evidenced by a rapid shrinkage of adipocyte [10]. However, the impact of leptin lowering for the metabolic adaptations after RYGB remains to be established.

Adiponectin levels are reduced in patients with type 2 diabetes compared with healthy controls. Experimental and epidemiological studies have suggested a role of adiponectin to promote and maintain insulin sensitivity and normoglycemia high levels indicate a lower risk for type 2 diabetes [26]. We observed that adiponectin levels did not change at 4 weeks but increased at 24 weeks post-RYGB, suggesting a possible contribution of this protein in glycemic control in the long term. This could be a consequence of adipose tissue gradually becoming more insulin sensitive over time [10, 27].

The circulating levels of BMP4 were higher at 24 weeks than the other two visits, and this can promote differentiation of preadipocytes into mature adipocytes [28]. Resistin is mainly secreted by immune cells in adipose tissue and has been associated with insulin resistance. Surprisingly, we found increased circulating levels of resistin 24 weeks after RYGB, which could be due to adipose tissue remodeling [29]. Likewise, visfatin levels were increased at 24 weeks and may have a role in regulating insulin sensitivity after RYGB.

### Cortisol and GH/IGF-1

It has been shown previously that ACTH levels are significantly increased 3 weeks after RYGB along with a concomitant increase of cortisol levels [30]. Cortisol levels were however reduced at 4 weeks after surgery in our study which may suggest a rapid and temporary attenuation of HPA axis activity, which could contribute to the initial insulin sensitization. RYGB has been previously shown to restore the GH/IGF-1 axis [31] and this was seen in our study with a significant rise at 24 weeks.

### Fatty acid turnover

Chronic elevation of NEFA levels has been reported in obese type 2 diabetes patients and can contribute to insulin resistance and beta-cell failure [32]. This could be due to increased fat mass and/or adipocyte size and might be explained by increased adipose tissue lipolysis or reduced lipid utilization or storage [32]. Non-diabetic obese individuals showed marked lipid mobilization at 1 month following RYGB, with twofold and sixfold elevation of NEFA and beta-hydroxybutyrate levels, respectively, despite increased insulin sensitivity [33]. We found no significant change in NEFA levels during fasting or OGTT at 4 weeks after RYGB, but lower levels at 24 weeks, compatible with improved insulin sensitivity with respect to lipid storage in adipose tissue. The unaltered glycerol levels suggest that lipolysis per se was not affected. These results are in agreement with our previous report [10] where the antilipolytic sensitivity of insulin studied ex vivo in isolated subcutaneous adipocytes did not significantly change at 4 and 24 weeks post surgery. In the present work a rise in NEFA between 120 and 180 min during OGTT was seen from 4 weeks after RYGB, as similarly observed in meal tests in non-diabetic post-GBP patients [34]. This rise in NEFAs may be due to the lower insulin levels in the operated individuals after 2 h following glucose ingestion.

### Autonomic nerve activity

It is known that the ANS plays a major role in body weight regulation, modulating satiety signals as well as energy expenditure [35]. This supports the theory that afferent vagal pathways are probably the most important link between the gut and the brain and interact with gut hormones. Our findings of increased heart rate variability, and in particular the HF component, suggest an elevated parasympathetic outflow after surgery following what has previously been shown [36]. In addition, the observed positive associations between the changes in HRV components with a change in insulin sensitivity indices at 4 weeks after RYGB may support a role of rapid changes in ANS activity for metabolic outcomes.

### Adipocytes

Adipocyte size has been shown as an independent risk factor for insulin resistance and reduction in fat cell size has been shown to be associated with improved insulin sensitivity at 1–2 years after RYGB [27]. We previously showed that at 4 weeks after RYGB adipocyte size was markedly reduced but, surprisingly, without changes in insulin action on adipocyte glucose uptake and lipolysis [10]. In the present analyses, we did not find any significant associations between HRV and hormones (GLP-1, GIP, GH, insulin, glucagon, and cortisol) at different time points post surgery with adipocyte size and adipocyte glucose uptake (data not shown). Interestingly, Matsuda index post surgery was increased before plasma adiponectin. This may suggest that the rapid improvement in insulin sensitivity after RYGB is mediated via extra-adipose pathways, involving gut, pancreas, adrenals, and pituitary as well as ANS. Adipose tissue might instead play a critical role in glycemic improvement later on. This is supported by the 24-week effects on adipocyte insulin sensitivity, adipokine levels, and also NEFA levels during OGTT [27].

### Limitations

We only investigated patients with type 2 diabetes and the sample size was small. Results should therefore not be extrapolated to other patient groups. The purpose of the control group was to evaluate other possible effects, by time or lifestyle adjustments and thus did not follow a matched diet for 4 weeks which would be an interesting comparison with the surgical group. Causality is not proven, and this will need specific interventions and detailed assessments in order to prove the role of neuroendocrine mechanisms. Another ongoing study by our group specifically compares the RYGB alone with LCD alone.

## Conclusion

Based on this hypothesis-generating study on the effects of RYGB in type 2 diabetes, we propose the following chain of events contributing to improved glycemic control. (1) An increased parasympathetic activity contributing to adaptive responses in the CNS. (2) Secondary effects on peripheral hormone regulation, including cortisol, glucagon and incretins, all of which are at least partly under nervous and neuroendocrine control. (3) Adipose tissue loss and functional changes. In the ongoing 2-year follow-up study, the time course and interplay of these events will be further elucidated.

Taken together, our findings support that neurohormonal mechanisms are involved in the rapid improvement following RYGB of glucose metabolism in type 2 diabetes. We hypothesize that shortly after surgery an elevated parasympathetic outflow and reduced cortisol axis activity may improve insulin sensitivity and act together with the increased incretin hormones to promote diabetes remission.

## Supplementary information

Supplementary material
