# Supplementary material for: Rapid changes in neuroendocrine regulation may contribute to reversal of type 2 diabetes after gastric bypass surgery
Source: Endocrine. 2020 Jan 26;67(2):344–53. doi: 10.1007/s12020-020-02203-w (PMC7026226; doi:10.1007/s12020-020-02203-w)
Supplement: Supplementary file 1 — Supplementary material [file 12020_2020_2203_MOESM1_ESM.docx]

**Electronic supplementary material**

## Biochemical measurements and Statistical analyses

Insulin, cortisol and insulin like growth factor -1 (IGF-1) were analyzed at the Department of Clinical Chemistry, Uppsala University Hospital. If not analyzed immediately, samples were stored at -80°C. Hormone analysis was done using commercially available kits. GH (Immulite 2000XPi; Siemens Healthcare Global), glucagon (Mercodia, Uppsala, Sweden) , total GLP-1 (Merck Millipore St Louise, MO, USA), total GIP (Merck Millipore, Darmstadt, Germany) and total Adiponectin (RnD Systems, Minneapolis, MN). Leptin, visfatin, resistin, and bone morphogenic protein 4 (BMP4) were analyzed using a magnetic bead-based Luminex assay (RnD Systems). The acute insulin response to arginine (AIRarg) was calculated using mean of the three highest plasma insulin concentrations obtained within 5 minutes after the arginine bolus minus the prestimulus plasma insulin concentration. Nonesterified fatty acids (NEFA) were measured using Free Fatty Acid Fluorometric Assay Kit (Cayman Chemical, Ann Arbor, MI). Glycerol using Free Glycerol reagent (Sigma-Aldrich, St. Louis, MO). The absorbance or fluorescence were read using a microplate reader (Infinite®200 PRO, Tecan, Männedorf, Switzerland).Top of Form

Bottom of Form

## Heart rate variability

A 5-minute resting HRV analysis, as previously described [1] , was performed to study the efferent activity in the autonomic nervous system. R–R intervals were extracted from the recorded ECG:s and transformed into evenly sampled (2 Hz) heart rate time series by cubic spline interpolation. HRV was analyzed by power spectrum analysis of R-R intervals related to normal sinus rhythm using Welch’s periodogram method as previously described [2]. The total spectral power, the power of the low-frequency (PLF,0.04-0.15 Hz), and high-frequency (PHF, 0.15-0.50 Hz), all log-transformed, were calculated over consecutive 5-minute periods from the complete recording. Since PHF mainly reflects the parasympathetic part while PLF reflects a combination of sympathetic and parasympathetic activity, the ratio PLF/PHF was used as a marker of the balance between sympathetic and parasympathetic activity [3]. The HRV analysis was performed using Matlab Software (MathWorks, Natick, MA).

## Statistical analyses

The sample size for this study was chosen to provide at least 80% power to detect HbA1c reduction by 1 % unit or more after RYGB surgery at 24 weeks, and this was based on data from previous studies [4]. The final sample size of 13 RYGB patients also allows more than 80% power to detect 15% changes in incretin and other hormones measured based on the results of previous data. We used linear mixed models with glucose, glycerol, NEFA, insulin, GLP-1, GIP, glucagon, IGF-1, growth hormone and adipokines, as outcome variables, and standard errors estimated by pairs-cluster bootstrap. As explanatory variables, we used categorical variables for the group, visit, OGTT-time point and all associated two- and three-way interactions.

Comparisons for AUC-values (baseline vs 4 weeks, baseline vs 24 weeks, 4 weeks vs 24 weeks) were performed using the Wilcoxon signed-rank test. Heart rate variability measures were compared using a paired t-test. The association between HRV variables and insulin sensitivity indices were analyzed by Spearman’s correlation.

Descriptive statistics are presented as a mean±standard deviation unless otherwise indicated. The area under the curve for OGTT-tests was calculated using the trapezoid method. All analyses were performed using Stata 14.

References

1. Svensson, M.K., Lindmark, S., Wiklund, U., Rask, P., Karlsson, M., Myrin, J., Kullberg, J., Johansson, L., Eriksson, J.W.: Alterations in heart rate variability during everyday life are linked to insulin resistance. A role of dominating sympathetic over parasympathetic nerve activity? Cardiovasc Diabetol **15**, 91 (2016). doi:10.1186/s12933-016-0411-8

2. Wiklund, U., Hornsten, R., Karlsson, M., Suhr, O.B., Jensen, S.M.: Abnormal heart rate variability and subtle atrial arrhythmia in patients with familial amyloidotic polyneuropathy. Ann Noninvasive Electrocardiol **13**(3), 249-256 (2008). doi:10.1111/j.1542-474X.2008.00228.x

3. Heart rate variability. Standards of measurement, physiological interpretation, and clinical use. Task Force of the European Society of Cardiology and the North American Society of Pacing and Electrophysiology. Eur Heart J **17**(3), 354-381 (1996).

4. Baskota, A., Li, S., Dhakal, N., Liu, G., Tian, H.: Bariatric Surgery for Type 2 Diabetes Mellitus in Patients with BMI <30 kg/m2: A Systematic Review and Meta-Analysis. PLoS One **10**(7), e0132335 (2015). doi:10.1371/journal.pone.0132335
